# Tainted Love: A Case of Gonococcal Endocarditis

**DOI:** 10.7759/cureus.26420

**Published:** 2022-06-29

**Authors:** Silpita Katragadda, Jennifer L Miatech, Ashley Zboril, Gift Echefu, Catalina Negulescu

**Affiliations:** 1 Internal Medicine Residency Program, Baton Rouge General Medical Center, Baton Rouge, USA; 2 Internal Medicine Residency Program, Baton Rouge Medical Center, Baton Rouge, USA; 3 Internal Medicine, Baton Rouge General Internal Medicine Residency, Baton Rouge, USA; 4 Internal Medicine, Baton Rouge General, Baton Rouge, USA

**Keywords:** valvular endocarditis, primary genitourinary gonococcal infection, endocarditis, disseminated gonococcal infection, gonococcal endocarditis

## Abstract

Gonococcal endocarditis (GE) is a rare complication of disseminated gonococcal infection with significant morbidity and increasing mortality despite early diagnosis and surgical intervention. The discovery and use of antibiotics in the treatment of gonococcal infections has made this once relatively common entity a rarity. Notably, over the recent years, GE has shown an alarming resurgence for unclear reasons. The upward trend in the incidence of gonococcal infections coupled with observed antibiotic resistance may in part explain the rise in cases. GE mostly affects sexually active young people without a history of valvular heart disease. Prompt diagnosis and surgical intervention are important in the bid to mitigate poor outcomes. Management is therefore multidisciplinary; primary care clinicians who usually are the first to see this patient population should be able to make an early diagnosis and facilitate early referral for surgical intervention as indicated. Despite appropriate and timely therapy, devastating consequences of this condition are not uncommon.

## Introduction

Gonorrhea is the second most common sexually transmitted disease in the United States with an 82.6% increase in the incidence rate reported since the historic low in 2009 [1] This implies that clinicians should expect a relative increase in the incidence of rare complications of disseminated gonococcal infection (DGI) including endocarditis. The presentation can be obscure without prior genito-urinary symptoms. Advancements in echocardiographic imaging, antibiotic susceptibility, and operative management play into the dynamics of this disease, and early recognition are important to prevent complications. We present a case of gonococcal endocarditis (GE) involving the mitral valve in a young male without a history of valvular heart disease.

## Case presentation

A 24-year-old male presented with a two-month history of fever, malaise, and lower extremity pain with associated weakness. He also complained of lethargy, chills, and myalgias. A review of systems was significant for night sweats, dyspnea on exertion, nausea, vomiting, decreased oral intake, and weight loss. He denied any paroxysmal nocturnal dyspnea, orthopnea, lower extremity swelling, hematuria, dysuria, or penile discharge. His family noted the patient had been relatively immobile, spending most of his time in bed since symptoms began. His past medical history was significant for polysubstance abuse but denied any intravenous drug use. The patient was admitted to unprotected sexual intercourse with a female prior to symptom onset. He was seen at an urgent care clinic sometime between the sexual encounter and his presentation to the emergency room. He was prescribed Ciprofloxacin 500 mg twice daily, for an unknown diagnosis which he did not take.

On initial presentation, he was noted to have an oral temperature of 98.8 F, a blood pressure of 90/62 mm Hg, a heart rate of 121 beats per min, a respiratory rate of 18 per min, and oxygen saturation of 99% in room air. Physical examination revealed a thin, malnourished, uncomfortable appearing young male. Skin examination was normal. Cardiac examination revealed a blowing systolic murmur heard best over the tricuspid area; no jugular venous distention was noted. There was no hepatosplenomegaly, pitting edema or joint line tenderness. He had decreased sensation to touch on the soles of his feet bilaterally. Laboratory data were significant for microcytic anemia with a hemoglobin level of 9.5 g/dL, leukocytosis at 22.77 K/µL with 85% neutrophils, and a platelet count of 93 micro/L. Troponin was elevated at 0.193 ng/dL. The urine drug screen was positive for cannabinoids and methamphetamine (Table 1). A 12-lead electrocardiogram revealed sinus tachycardia with a rate of 121 bpm, with no ST or T-wave abnormalities.

**Table 1:** Laboratory findings

Laboratory Findings		Reference range
Hemoglobin	9.5 g/dL	13.7-17.0 g/dL
WBC	22.77 K/µL (85% neutrophils)	4.0-11.0 K/µL
Platelet count	93	150-400 K/µL
Troponin	0.193	<0.015 ng/dL
Urine drug screen	Positive cannabinoids and methamphetamine	

**Figure 1:**
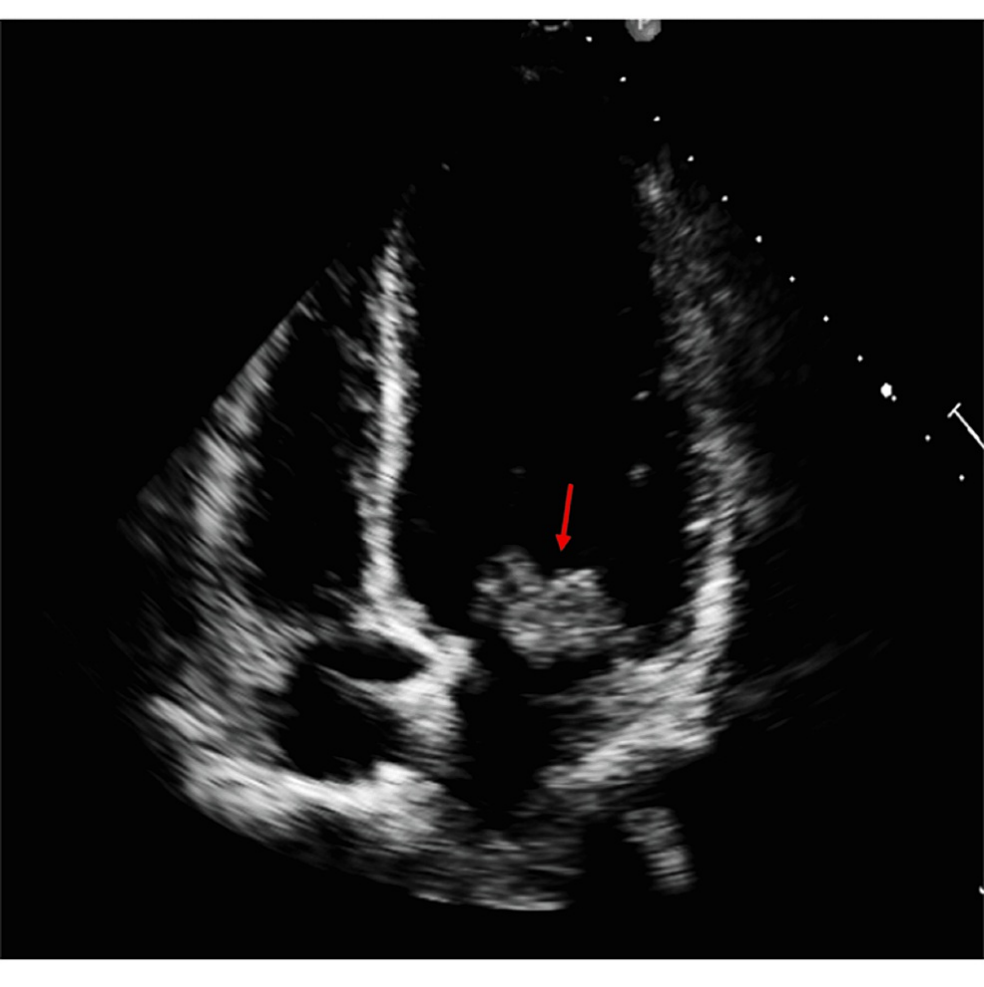
Parasternal long axis showing mitral valve vegetation (red arrow)

**Figure 2:**
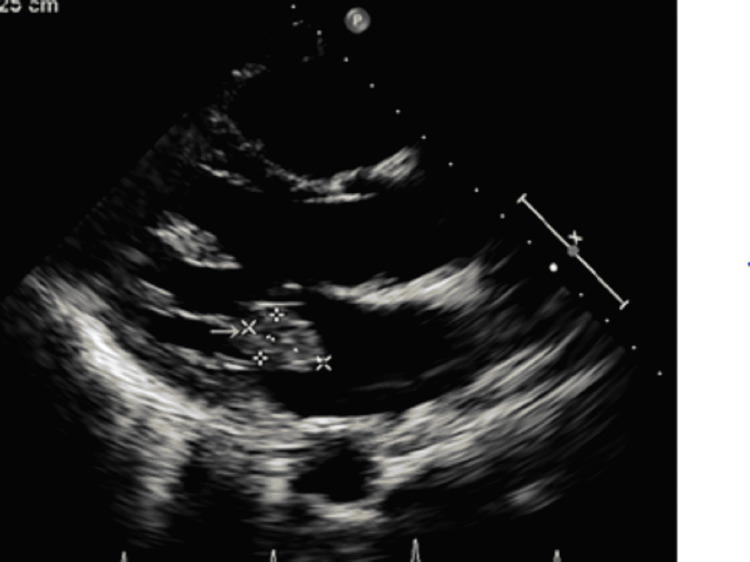
Parasternal short axis view showing mitral valve vegetation (yellow arrow)

Blood cultures were obtained, and the patient was initiated on empiric antibiotics; ceftriaxone 1 g daily and vancomycin 15mg/kg q 12 hours. A transthoracic echocardiogram was performed showing a 3.5 x 2.5 cm echogenic mass attached to the anterior and posterior leaflets and medial corner of the mitral valve with severe mitral regurgitation and mitral valve prolapse (Figures 1, 2). On the third day of admission, three sets of blood cultures drawn in a span of 4 hours on the day of admission were reported positive for Neisseria gonorrhoeae with DNA probe confirmation. The bacteria in all cultures were sensitive to ceftriaxone, negative for beta-lactamase screening.

**Figure 3:**
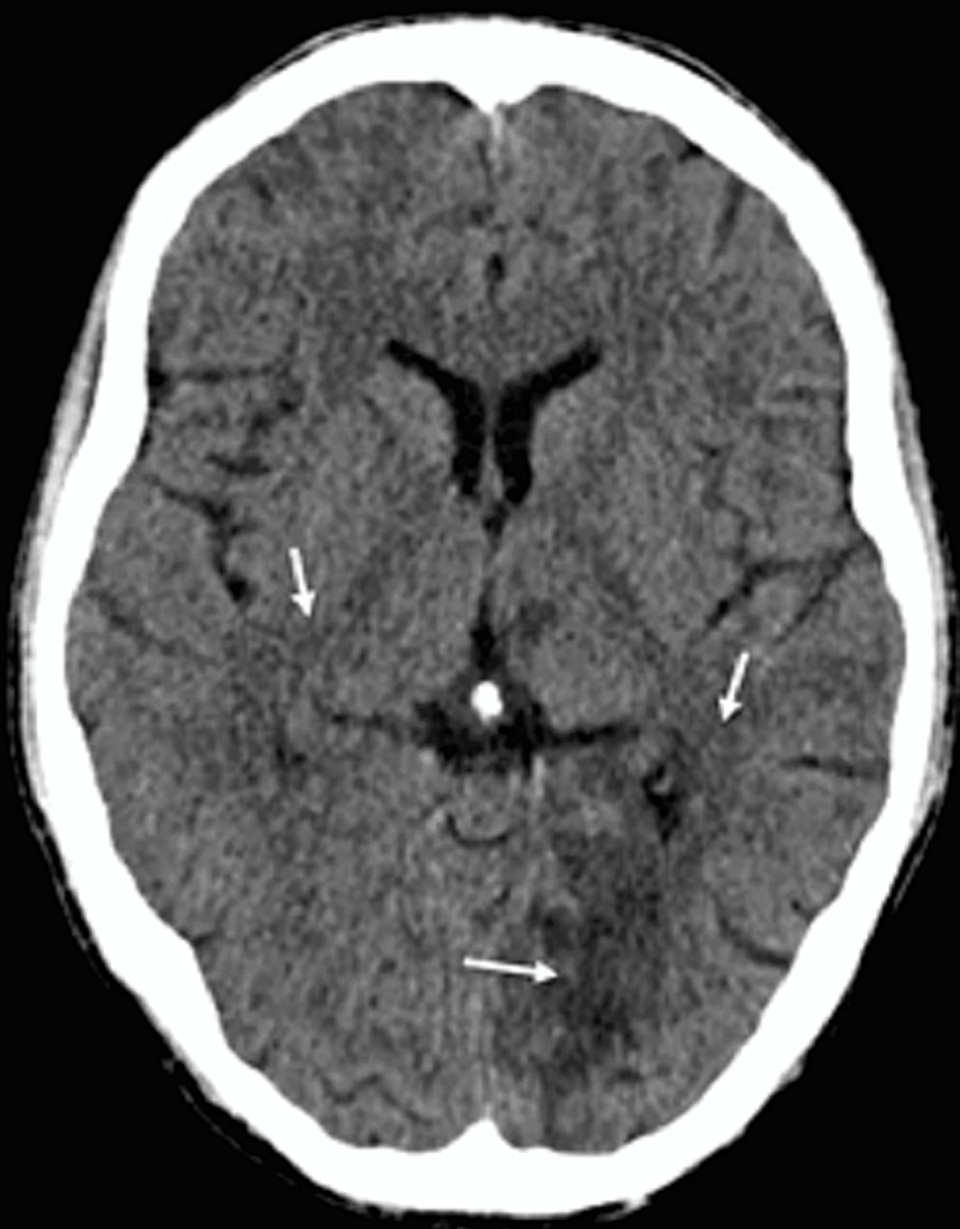
Head CT without contrast showing multifocal infarctions (white arrows)

**Figure 4:**
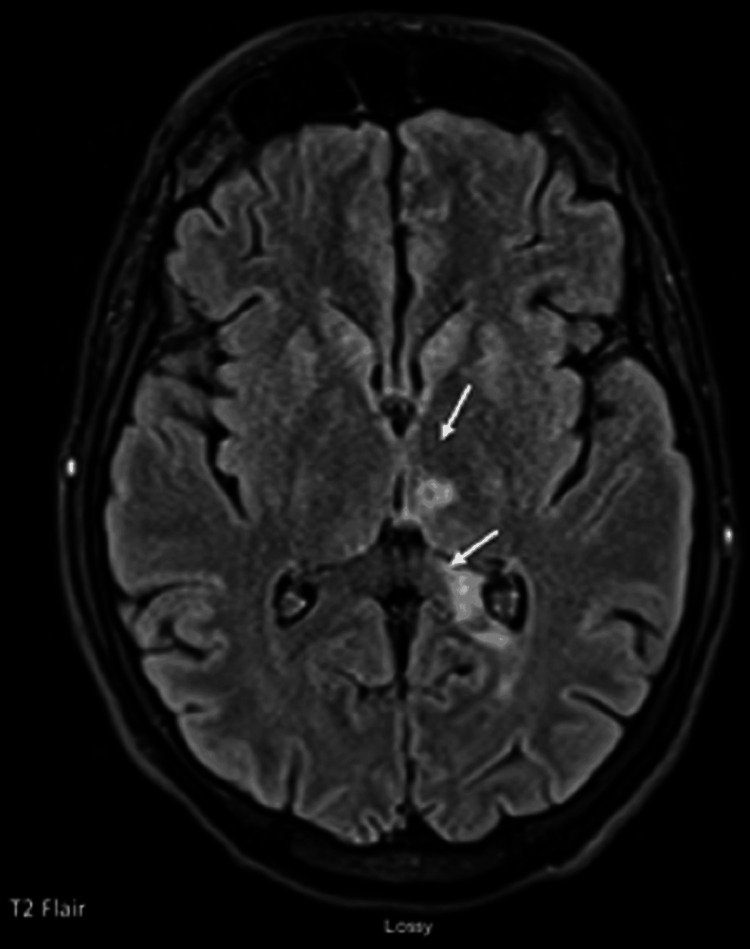
MRI of the head without contrast showing multifocal infarctions (white arrows)

Cardiovascular surgeon was consulted for possible mitral valve replacement. However, on the morning of hospital day 3, the patient developed acute onset right-sided hemiparesis and aphasia. CT scan of the head demonstrated subacute left thalamic and left occipital lobe territory infarct (Figure 3). Subsequent MRI brain revealed multiple infarcts in the left occipital, left thalamic, left lentiform nucleus, left corona radiata, and right corona radiata (Figure 4).

He subsequently underwent a successful mitral valve replacement, tissue culture confirmed N. gonorrhea, and antibiotics were de-escalated to ceftriaxone. He continued to improve on intravenous ceftriaxone. His neurological deficits improved with aggressive physical therapy during the remainder of the admission. Ultimately, the patient was transferred to a long-term acute care facility to continue physical therapy and completed six weeks course of IV ceftriaxone. Surveillance cultures drawn on days 2, 9 and 23 after admission remained negative.

## Discussion

Gonorrhea remains the second most reported sexually transmitted infection in the United States, contributing to the public health burden since medieval times. It is seen in a marginalized population of society, mostly afflicting the poor, sexually active young population, and newborns. The Centers for Disease Control and Prevention estimated approximately 1.6 million new infections occurred in the United States in 2018.

In the pre-antibiotic era, a significant portion of bacterial endocarditis (up to 26%) was caused by gonococci [1-8]. The prognosis was usually dismal with supportive therapy being the mainstay of treatment. With the advent of antibiotic therapy, there has been a substantial decrease in endocarditis infections [9]. Even though gonorrhea remains prevalent in today’s world, the outcomes are significantly better and survival rates have risen. This was demonstrated in a report of 26 patients treated with antibiotics [8]. A systematic review of 25 years reported a series of GE that occurred after 1983. This finding was surprising because no cases were detected in the preceding 20 years. The reason for this resurgence in the later part of the 20th century and the decline in the 21st century is unclear [8].

Gonorrhea is commonly seen in younger people with an age range of 15-35 years with male predominance. Overall, about 1-3% of patients develop DGI, and women appear to be at a higher risk [1]. This may be attributable to the high number of asymptomatic infections in women. Disseminated infection is also observed to rarely affect pregnant and menstruating women, although there has been no proven association with endocarditis [10-12].

The gonococcal infection has a wide range of clinical manifestations from asymptomatic infection, and isolated genitourinary infection, to disseminated infection involving the skin or joints. Rarely, the disseminated infection may present as endocarditis, estimated to occur in 1%-2% of cases [2]. It has been reported to affect both males and females equally [7]. Primary genitourinary gonococcal infection occurs in 21%-27% of GE cases, either concomitantly or months prior to the diagnosis of endocarditis [13].

The clinical manifestations of GE are similar to endocarditis caused by other pathogens. Presentation typically includes the presence of fever, tachycardia, cardiac murmur, and signs or symptoms of acute heart failure. Diagnosis of GE is often established with blood cultures, which are positive in more than 90% of cases. This differs from other forms of DGI where blood culture positivity is only 10% and 30% [13]. Laboratory findings commonly present in endocarditis include anemia, leukocytosis, and elevated inflammatory markers although these tests are not specific for GE. Diagnosis of endocarditis is confirmed following a transthoracic or esophageal echocardiogram [14]. The aortic valve is most commonly affected.

Although it is challenging to differentiate the etiology of endocarditis based on clinical presentation, there are some attributes that can point toward a gonococcal etiology. In comparison to streptococcal and staphylococcal endocarditis, GE has a propensity to affect younger patients with healthy native valves, especially the aortic valve [13]. The period between the outbreak of symptoms and detection of disease also tends to be longer [2,4] compared to pneumococcal and staphylococcal disease. These patients may also have a dramatic progression to heart failure. Another contrasting feature is the lack of known previous local gonococcal infection compared to patients with endocarditis caused by gram-positive organisms, in whom a gateway of entry is often identified [7]. 

Treatment involves parenteral antibiotics and often cardiac valve surgery. The Centers for Disease Control recommends parenteral antimicrobial therapy with ceftriaxone for at least four weeks [15]. Ultimately, antibiotic management should be guided by the results of antimicrobial susceptibility testing in consultation with an infectious-disease specialist. The majority of patients with GE require valve surgery alongside antibiotics therapy, which is usually as a result of worsening congestive cardiac failure. Several case reports on management of mitral valve GE have reported improvement and survival with a combination of antibiotics and valve replacement (Table 2) [16-18]. Third-generation cephalosporins (ceftriaxone or cefotaxime) for four to six weeks were mostly prescribed by the authors (Table 2). Valvular surgery recommendations are similar to other forms of left-sided native valve endocarditis, including valve dysfunction causing symptoms or signs of heart failure, a paravalvular extension of infection with development of an annular or aortic abscess, destructive penetrating lesion, heart block, and persistent infection (manifested as persistent bacteremia or fever lasting more than seven days after initiation of appropriate antibiotic therapy). Early surgery, within the first week of antibiotic therapy, may be considered to reduce the risk of embolism in patients with large vegetations (>10 mm). The mortality rate of GE is strikingly high at 19%, despite appropriate antibiotics and surgical intervention [1].

**Table 2:** Comparison of case reports on mitral valve gonococcal endocarditis

Case report	Year reported	Complications	Management	Outcome
Harvatin et al. [16]	2022	Perforation and severe regurgitation	Ceftriaxone, Mitral valve replacement	Survived
Shetty et al. [2]	2004	severe mitral regurgitation	Gentamicin and Benzylpenicillin, then Rifampin and cefotaxime, Mitral valve replacement	Survived
Kenth et al. [17]	2020	Mild stenosis and moderate regurgitation	Ceftriaxone, Mitral valve replacement	Survived
Butterly et al. [18]	2011	Mitral regurgitation, mycotic aneurysm of the superior mesenteric artery	Ceftriaxone, Mitral Valve replacement, Superior mesenteric aneurysmal repair	Survived
Present case	2022	Embolic (septic) acute cerebrovascular	Ceftriaxone, Mitral valve replacement	Survived

DGI presenting as endocarditis remains fairly uncommon (Table 2). Our patient presented with symptoms of endocarditis, which were initially presumed to be from more common bacterial infections, such as staphylococcus endocarditis given his known polysubstance abuse history. The patient’s recent high-risk sexual encounter provided exposure. Subsequently, blood cultures and transesophageal echocardiogram confirmed a diagnosis of GE revealing large mitral valve vegetation. The large vegetation size prompted urgent surgical evaluation, unfortunately, the patient developed an embolic acute cerebrovascular incident prior to surgery. Following mitral valve surgery and prolonged antibiotics, the patient demonstrated dramatic improvement in his aphasia and right-sided hemiparesis.

## Conclusions

The poorly treated gonococcal infection has the potential of progressing to DGI, with GE is a rare but potentially fatal complication. A low threshold for suspicion and timely management with guideline-directed therapy is required to prevent this catastrophe. Therefore, in a patient failing to improve following appropriate management of uncomplicated gonorrhea, reassessment is required to ensure eradication, as high resistance rates of the bacteria to antibiotics are an established problem. It might be necessary to counsel and re-test patients three to 12 months after infection due to high re-infection rates.
